# Imaging diagnosis of metastatic breast cancer

**DOI:** 10.1186/s13244-020-00885-4

**Published:** 2020-06-16

**Authors:** Filippo Pesapane, Kate Downey, Anna Rotili, Enrico Cassano, Dow-Mu Koh

**Affiliations:** 1 Breast Imaging Division, IEO - European Institute of Oncology IRCCS, Via Giuseppe Ripamonti, 435, 20141 Milano, MI Italy; 2 Department of Breast Radiology, Royal Marsden Hospital, Downs Road, Sutton, SM2 5PT UK; 3 Cancer Research UK Cancer Imaging Centre, The Institute of Cancer Research, 15 Cotswold Road, Sutton, SM2 5NG UK; 4 Department of Radiology, Royal Marsden Hospital, Downs Road, Sutton, SM2 5PT UK

**Keywords:** Bone metastases, Breast cancer, Cancer staging, Oncology, Positron-emission tomography, Radiology

## Abstract

Numerous imaging modalities may be used for the staging of women with advanced breast cancer. Although bone scintigraphy and multiplanar-CT are the most frequently used tests, others including PET, MRI and hybrid scans are also utilised, with no specific recommendations of which test should be preferentially used. We review the evidence behind the imaging modalities that characterise metastases in breast cancer and to update the evidence on comparative imaging accuracy.

## Key points

1. Combined local- and whole-body staging is crucial for breast cancer treatment.
2. Bone scintigraphy is easy to read, widely available and cost effective.
3. Whole-body-MRI/hybrid imaging is increasingly performed for distant staging.
4. PET-CT may detect metastases with higher sensitivity than conventional imaging.
5. WBMRI also plays a role in the detection of visceral and skeletal involvement.

## Introduction

Due to the rising incidence of breast cancer, it is estimated that breast cancer-related deaths will increase by 43% globally from 2015 to 2030 [1]. At presentation, 4–10% of breast cancers are metastatic [2] and accurate staging of breast cancer is crucial for guiding treatment and optimising patient outcome. Imaging provides information regarding the presence, extent and distribution of metastatic disease. The aim of this review is to critically evaluate the current imaging techniques for the detection of metastatic disease in breast cancer, highlighting the new and emerging methods to optimally stage patients with advanced disease.

### Literature search strategy and selection criteria

Using a software based on statistical text mining and machine learning methods [3], we identified 33 relevant articles in PubMed on staging of advanced breast cancer, metastatic breast cancer, bone disease and distant metastases between 2009 and 2019. We identified papers in English language, both original studies and review articles. Additionally, we searched the references listed for additional relevant papers, using a total of 40 scientific papers.

### Staging of breast cancer for metastatic disease

The most common staging system for breast cancer is the American Joint Committee on Cancer (AJCC) TNM [4], which is based on tumour size and the degree of locoregional invasion by the primary tumour (T), the extent of regional lymph node involvement (N) and presence (or absence) of distant metastases (M) [5] (Table 1). M1 indicates the presence of any metastases to distant organs, implying a stage IV disease (regardless of the T or N status). Breast cancer may be stage IV at first diagnosis, or it can be recurrent from previous breast cancer. Stage IV disease showed a 5-year survival rate of approximately 22%, although this rate varies according to other factors, such as the hormone receptor status [6]. The median survival for patients with breast cancer and bone metastases is 65 months in the oestrogen/progesterone-receptor-positive (ER/PR-positive) groups, and 40 months in both the human epidermal growth factor receptor 2 (HER-2) positive and ‘triple-negative’ group [7].

**Table 1:** Breast cancer staging

Stage	T	N	M	Description
0	Tis	N0	M0	Tumour that has not grown beyond its site of origin and invaded the neighbouring tissue. It includes the DCIS and LCIS.
IA	T1 (tumour ≤ 20 mm)	N0	M0	Tumour which is not ‘in situ’ but it is ≤ 20 mm in greater dimension
IB	T0 or T1	N1mi (micrometastases)	M0	Tumour ≤ 20 mm in greater dimension with nodal micrometastasis (greater than 0.2 mm and/or more than 200 cells, but none greater than 2 mm)
IIA	T0 or T1	N1 (metastases in 1–3 ipsilateral ALN(s)	M0	Tumour ≤ 20 mm in greater dimension with involvement of axillary lymph nodes or tumour from 20 to 50 mm without involvement of any ALNs
	T2 (20 mm < tumour ≤ 50 mm)	N0	M0	
IIB	T2	N1	M0	Tumour from 20 to 50 mm with involvement of ALNs or tumour > 50 mm without involvement of any ALNs
	T3 (tumour > 50 mm	N0	M0	
IIIA	T0, T1 or T2	N2 (metastases in 4–9 ipsilateral ALNs)	M0	Tumour > 50 mm with spread to ALNs, or tumour of any size with metastases in ALNs which are knitted to each other or with the surrounding tissue
	T3	N1 or N2	M0	
IIIB	T4 (tumour of any size with direct extension to the chest wall and/or to the skin	N0, N1, N2	M0	Tumour of any size with metastases into the skin, chest wall or internal LNs of the mammary gland
IIIC	Any T	N3 (metastases in ≥ 10 ALNs, or in infra-clavicular LNs or ipsilateral internal mammary LNs)	M0	Tumour of any size with a more widespread metastases and involvement of more LNs
IV	Any T	Any N	M1 (distant organs’ metastases)	Any tumour spreads to parts of the body that re located far removed from the chest (bones, lungs, liver or distant LNs)

*ALN* axillary lymph node, *DCIS* ductal carcinoma in situ, *LCIS* lobular carcinoma in situ, *LN* lymph node

• T2, T3 and T4 tumours with nodal micrometastases (N1mi) are staged using the N1 category

• M0 means that there are no clinical or radiographic evidence of distant metastases. It includes also M0(i+) that indicates the presence of tumour cells or deposits < 0.2 mm detected microscopically or by molecular techniques in circulating blood, bone marrow or other nonregional nodal tissue in a patient without clinical and radiographic evidence of distant metastases

• Stage 3a is broadly known as a local spread of breast cancer

• T4 does not include the invasion of dermis alone

• If a patient presents with M1 disease prior to neoadjuvant systemic therapy, the stage is considered stage IV and remains stage IV regardless of response to neoadjuvant therapy

• Stage designation may be changed if postsurgical imaging studies reveal the presence of distant metastases, provided the studies are performed within 4 months of diagnosis in the absence of disease progression, and provided the patient has not received neoadjuvant therapy

Multi-modality imaging is widely used clinically for disease staging. However, all cancers are potentially systemic diseases and whole-body imaging techniques, such as whole-body hybrid imaging (PET-CT and/or PET-MRI) or whole-body magnetic resonance imaging (WBMRI) are increasingly performed to reflect this.

### The approach to the patient with suspected advanced breast cancer

There are now effective lines of treatment for patients with metastatic disease, which can improve symptoms, prevent complications and prolong life [8–13]. Furthermore, oligometastatic disease (typically < 5 metastases), may be suitable for aggressive local therapy in combination with systemic treatment. Hence, the combined assessment of local disease and whole-body staging, together with better understanding of the tumour molecular characteristics, is key to individualised treatment [14, 15].

The detection of breast cancer metastases in breast cancer varies according to disease stage. In early breast cancer, routine staging evaluations are directed at locoregional disease [16] as in stages T1 and T2 primary breast cancers, the incidence of distant metastases is < 2% [17] compared with 15–20% in stage T3 or T4 [18]. Accordingly, the American Society of Clinical Oncology (ASCO), the European Society for Medical Oncology (ESMO) and the Royal College of Radiologists (UK), in their clinical practice guidelines for breast cancer (updated in 2018, 2019 and 2014, respectively), do not recommend routine imaging for the M-staging of asymptomatic patients with early stage disease. Staging imaging studies are usually performed for patients at high risk of disease spread, such as stage T3/4 cancers (> 5 cm), in patients with 4 or more involved axillary lymph nodes or in the setting of recurrent disease [16, 19].

The incidence of bone metastases in ER/PR positive cancer, which may establish upwards of 10–20 years after initial diagnosis [20], is significantly higher than the incidence of other site of metastases, namely is reportedly 18.7% (in luminal A subtype) to 30.4% (in luminal B subtype) [21]. Furthermore, the molecular subtype of the breast cancer influences the likelihood of metastatic spread: women with ER/PR-negative tumours have a higher risk of metastatic relapse in the first 5 years [20, 21] compared with ER/PR-positive tumours.

Current international guidelines lack consensus as to whom and how to image for metastatic disease [22]. Traditionally, high-risk patients were screened for occult metastases using bone scintigraphy (BS), chest radiography and abdominal ultrasound or bone scintigraphy and CT of the chest abdomen and pelvis. However, the use of next generation imaging, such as hybrid imaging (PET-CT and/or PET-MRI) and WBMRI, has increased over the years [17]. Indeed, traditional conventional imaging frequently detects bone disease and visceral metastases in late stages, which are associated with poorer outcomes. Moreover, these methods often fail to demonstrate the heterogeneity of the tumour biology, leading to delay in the detection of treatment resistance and the opportunity for therapeutic modifications [15].

The ESMO’s guidelines recommend performing chest, and abdominal imaging (US, CT or MRI scan) and a bone scan can be considered for patients with clinically positive axillary nodes, large tumours (T3/4) or tumours with aggressive biology. If such methods are inconclusive, dual imaging methods combining functional and anatomical information such as ^18^F-FDG PET-CT are suggested [16].

The Royal College of Radiologists (UK) recommends staging with CT of the chest abdomen and pelvis for patients with large (T4) tumours or with heavy lymph node burden (N2 disease) with or without bone scan and a PET-CT for suspected inflammatory breast cancer.

The latest North American National Comprehensive Cancer Network (NCCN) guidelines [23] recommend BS, abdominal CT/MRI (including the pelvis if symptomatic), chest CT/^18^F-NaF PET-CT, in symptomatic patients or in stage I-IIB breast cancer and abnormal liver function test, elevated serum alkaline phosphatase, localised bone pain. The ^18^F-FDG PET-CT is often recommended when the findings of conventional imaging are suspicious or uncertain. BS or ^18^F-NaF PET-CT may be bypassed when ^18^F-FDG PET-CT has already detected skeletal metastasis. Other requests for imaging may result from multi-disciplinary team discussions, e.g. in patients with triple negative invasive carcinoma, or in ipsilateral recurrence within the breast [24].

Regarding bone metastases, every above-mentioned imaging modality evaluates different aspects of the tumour: BS estimates osseous remodelling and osteoblastic activity, CT reveals bone destruction and/or presence of sclerosis, diffusion-weighted MRI assesses tissue cellularity and PET-CT using FDG tracer evaluates increased glycolytic metabolism [25]. Conventional imaging is limited when detecting small bone metastases: BS may have an unsatisfactory performance for lytic lesions, metastases with low bone turnover and low vascularity. CT usually demonstrates lytic lesions associated with bone destruction, but disease confined to the bone marrow may be missed [25].

In the next section, we review the diagnostic utility of conventional imaging (BS, CT and MRI) and next generation imaging (PET-CT, PET-MRI and WBMRI) for assessing the presence, extent and biological characteristics of bone and visceral metastases in patients with breast cancer.

## Conventional imaging

### Radionuclide bone scan

#### Planar bone scintigraphy (BS)

Planar BS is clinically easy to read, widely available and cost effective [26], and has been recommended as the primary technique to detect bone metastases in asymptomatic high-risk breast cancer women [17].

Abnormal accumulation of ^99m^Tc-labelled diphosphonates is connected to increased local blood flow and osteoblastic activity which occur consequently to metastatic growth within bone marrow [17, 27–30]. The sensitivities of planar BS reportedly range between 62 and 100% [17, 27–31]. However, ^99m^Tc-labelled diphosphonates are nonspecific markers of osteoblastic activity which are also observed in benign fractures, Paget’s disease, degenerative joint diseases, trauma and inflammation [31–33]. This accounts for the lower specificity of planar BS (0.75, 0.71–0.79) than SPECT (0.85, 0.80–0.90) (Table 2) [29, 34–36].

**Table 2:** Studies comparing diagnostic performance of BS and SPECT

Ref	First author	Year	Patients/lesions	Study design	Sensitivity of BS (vs. SPECT)	Specificity of BS (vs. SPECT)
[29]	Shen	2014	N/R	Met-analyses	59% (vs. 90%)	745 (vs. 85%)
[34]	Giovanella	2011	194/245	Prospective	75% (vs. 95%)	74% (vs. 82%)
[35]	Nozaki	2008	39/116	N/R	N/R	69% (vs. 90%)
[36]	Palmedo	2014	211/353	Prospective	93% (vs. 94%)	77% (vs. 95%)
[37]	Even-Sapir	2006	44/156	Prospective	70% (vs. 92%)	57% (vs. 82)
[38]	Khalil	2011	N/R	Systemic review	N/R	74% (vs. 94%)

#### SPECT bone scan

**Fig. 1:**
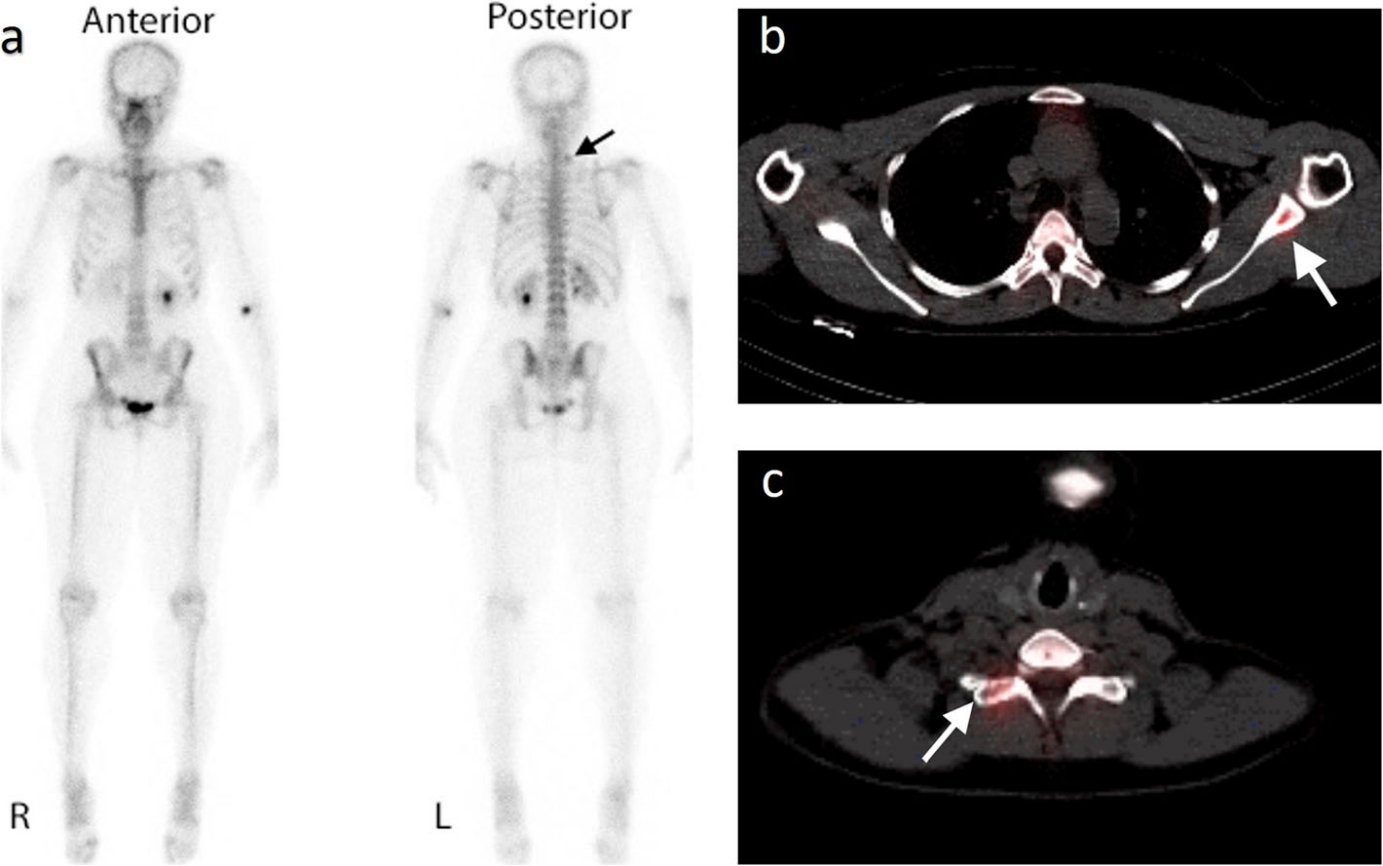
Planar bone scan versus SPECT. Images from a planar bone scan (**a**) and views from a single-photon emission computerised tomography (SPECT) through the thoracic (**b**) and cervical (**c**) spine in a 56-year-old woman with breast cancer. Subtle abnormal tracer accumulation is present at the root of the left neck on the planar bone scan (black arrow) with normal accumulation within the urinary tract and at the injection site (**a**). SPECT-CT demonstrates corresponding uptake in right transverse process of C7 (**c** white arrow) but additional uptake in the left scapula (**b** white arrow) not visible on the planar images alone

An overall improvement was observed when BS was augmented using single-photon emission computerised tomography (SPECT) in the lower thoracic and lumbar spine, or across the entire axial skeleton (whole-body SPECT) (Fig. 1) [37, 39–41]. On a per-patient basis in breast cancer, SPECT showed a higher specificity of 94% compared with 74% using BS [42]. Although some pre-clinical SPECT scanners can provide a sub-millimetre spatial resolution, clinical gamma cameras yield a tomographic resolution of about 10 mm [38]. Hence, like planar BS, the spatial resolution of SPECT is limited [41]. Accordingly, the diagnostic effectiveness of SPECT has been questioned and patients with uncertain findings often demand other radiological exams to characterise indeterminate findings [43–45].

Combining SPECT with CT partially improves the performance for metastatic bone disease, by lowering the number of equivocal lesions detected on planar BS [26, 36, 46]. However, such combined technique may not be widely available because of higher equipment costs, and image quality may be reduced by patient movement and CT artefacts [26].

#### CT

In clinical practice, plain radiographs are often used as an adjunct to BS, to evaluate symptomatic bone pain. Bone scan findings are often corroborated using CT [17], which shows a good specificity although a poor sensitivity (95% and 73% respectively, according to Heindel et al.) [47]. Metastases appear on CT as areas of lucency or sclerosis, and rarely as well-defined radiodensity within the bone marrow [21].

Concerning visceral metastases, liver localisations are quite common in patients with breast cancer (up to 60–70% of women have liver metastases at autopsy) [48]. Sensitivity and specificity for detecting liver metastases were reported 73–75% and 94–96%, respectively, while for US, MRI and FDG-PET were 61–65% and 96–98%, 80–82% and 96–98% and 94–96% and 98–99%, respectively [49–51].

**Fig. 2:**
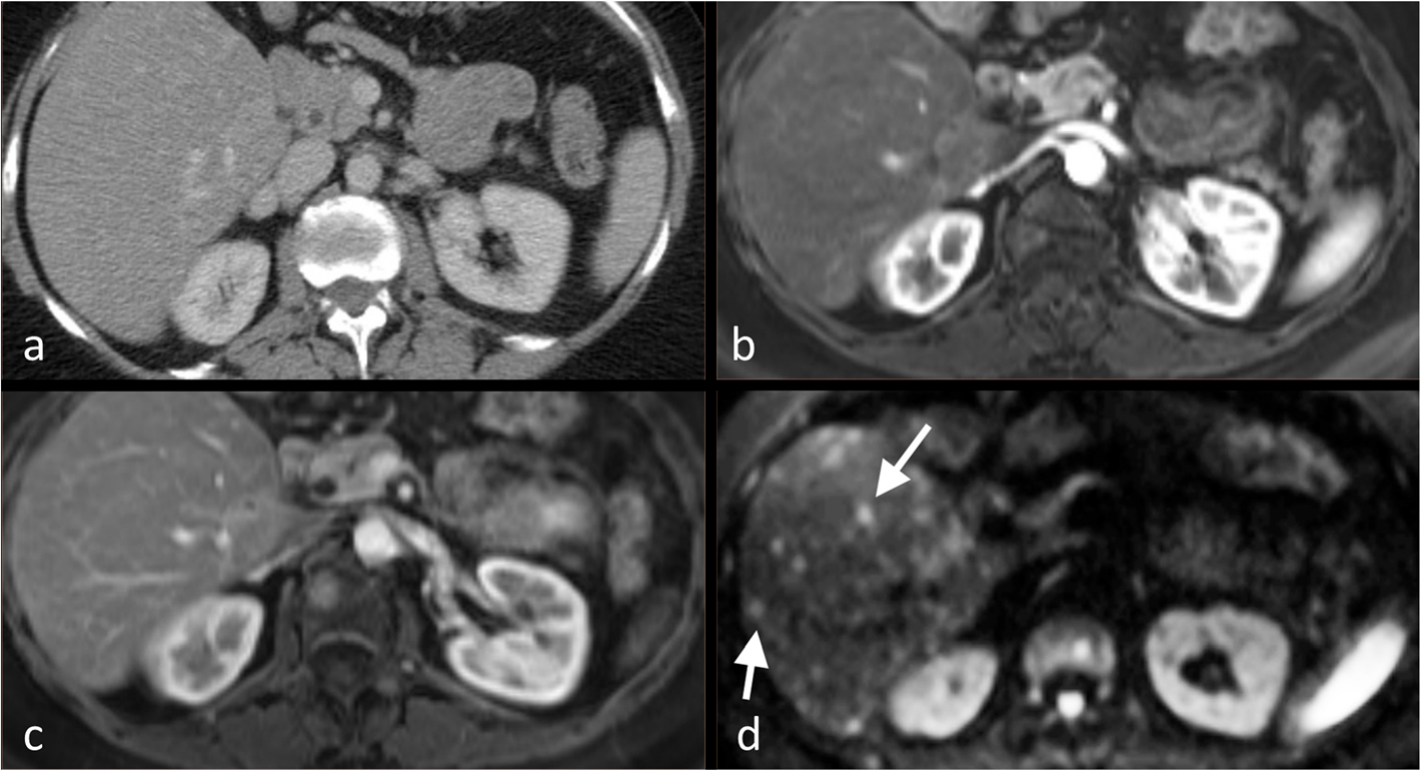
Isodense liver metastases not visualised on CT identified using MRI. Images from a portal venous phase CT (**a**), arterial (**b**) and portal venous (**c**) phase T1-W post-contrast MRI and b-750 diffusion-weighted MRI (**d**) through the liver in a 46-year-old woman with breast cancer and deranged liver function. The liver appears normal on both CT and T1-W post-contrast MRI but multiple high signal foci are present throughout the liver (white arrows) on the diffusion-weighted sequence in keeping with diffuse liver metastases

Generally, HER2-expressing breast cancers tend to metastasise to the liver more frequently than ER/PR-positive breast cancers [52, 53]. Portal venous phase of CT shows liver metastases as irregular hypodense lesions with peripheral contrast enhancement [54]. Nevertheless, liver metastases may appear isodense with the hepatic parenchyma (Fig. 2) and their extent may be underestimated or, when diffuse, entirely missed [21]. No differences were observed in the CT appearances of liver metastases according to the molecular hormonal receptor status [55].

Similarly, higher percentages of brain metastases are found in hormone ER/PR-negative breast cancers when compared with the hormone-positive subtypes [52]. Moreover, triple negative breast cancer subtype shows higher risk of developing brain metastases [56]. Performing CT of the brain is commonly induced by clinical symptoms such as mental status change, headache or vomiting/nausea. In this setting, MRI is preferred, where and when available, due to its enhanced sensitivity [21]. From the radiologist’s perspective, although innovative treatment may prolong overall survival, a higher rate of cerebral metastases is reported in such patients, as novel therapeutics may not be effective across the blood-brain barrier [57].

**Fig. 3:**
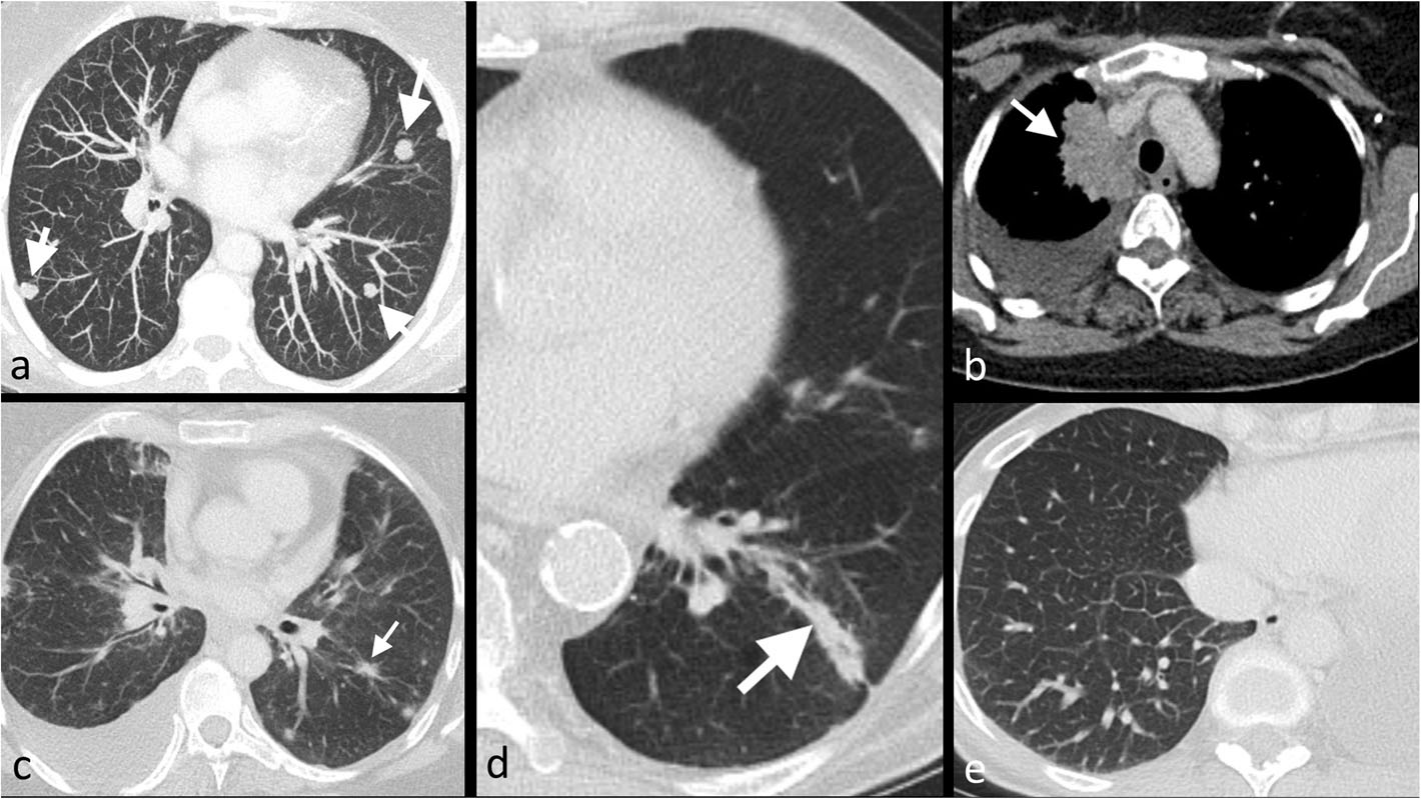
Morphological appearances of lung metastases. Maximum intensity projection (MIP) CT thorax (**a**), and portal venous phase CT thorax using mediastinal windows (**b**) and portal venous phase CT on lung windows (**c**, **d** and **e**) in a 62-year-old woman with differing morphological appearances of metastatic breast cancer to the lungs. The MIP CT demonstrates multiple rounded lung metastases (**a**), a mass lesion simulating lung cancer (**b**), irregular spiculated metastases (**c**), endo- and peribronchial infiltration (**d**) and lymphangitis carcinomatosis (**e**)

Lung metastases from breast cancer may be showed on CT in a number of radiological patterns: singular or multiple pulmonary nodules, endobronchial localisation, air-space consolidation and lymphangitic carcinomatosis, which may simulate other primaries such as pulmonary adenocarcinoma or lymphoma [21] (Fig. 3). Pleural localisation frequently reveals as a unilateral (and ipsilateral to the breast cancer) pleural effusion, with no specific features compared with benign effusions [21].

### Conventional MRI

In metastatic breast cancer, malignant cells displace and replace normal bone marrow fat cells causing a reduction in fat content, whereas successful management is associated with return of healthy bone marrow fat [58]. MRI can differentiate healthy from pathological bone marrow acting as an effective and non-invasive test to recognise bone metastases [17, 28, 59, 60]. MRI sequences including T1WI, diffusion-weighted imaging (DWI) and Dixon quantitative chemical shift imaging (which estimates water and fat fraction) can evaluate the anatomical and functional features of bone marrow [58, 61]. Therefore, a trained radiologist can discriminate bone metastases (which manifest with nodular focal metastatic lesions or marrow infiltration/replacement) from benign marrow alterations (such as marrow hyperplasia induced by chemotherapy) [28].

Studies evaluated MRI as the primary screening technique for diagnosing skeletal deposits to associate the advantages of high sensitivity and specificity with improved spatial resolution, demonstrating the diagnostic superiority of MRI over BS for identifying bone metastases [62, 63]. Particularly, an updated meta-analysis showed a pooled sensitivity of 97% for MRI versus 79% for BS and a pooled specificity of 95% versus 82%, respectively [29].

Additionally, MRI is commonly used to clarify equivocal findings from BS [28] and to detect complications associated with bone metastases (such as compression of spinal cord or nerves), which can alter management decisions [64]. Altehoefer et al. [65] showed how MRI guided the need for local therapy in sites that were negative on BS; Kim et al. [66] highlighted the value of MRI for the evaluation of single ‘hot spots’ on BS [67]. However, interpretation of bone MRI needs an understanding of the evolution of normal bone marrow with age: indeed in younger patients, highly cellular hematopoietic marrow may make it more difficult to diagnose small metastases [68].

**Fig. 4:**
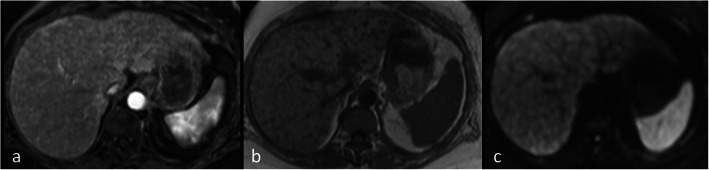
Hypervascular liver metastases on MRI. Liver MRI in a 52-year-old woman with breast cancer demonstrates multiple slightly T1 hyperintense lesions in the liver on the T1-W image (**a**) which show increased enhancement in portovenous phase (**b**) and impeded diffusion on b750 DWI (**c)**

With an 80–82% and 96–98% reported ranges of sensitivity and specificity, respectively [49–51], MRI is an appropriate modality to detect hepatic metastases [69]. Breast cancer metastases in liver are typically hypo- to isointense on T1WI, iso- to hyperintense on T2WI and, since breast metastases are frequently hypovascular, they usually show perilesional enhancement [70] in the arterial phase of contrast enhancement. However, hypervascular metastases may also occur, which can be associated with disease progression (Fig. 4). In addition, DWI can aid the detection of small liver metastases easily overlooked on other sequences [69]. Pretreatment enhancement features of liver metastases at MRI, such as the degree of hypervascularity of the tumour rim, may also predict disease progression [71].

**Fig. 5:**
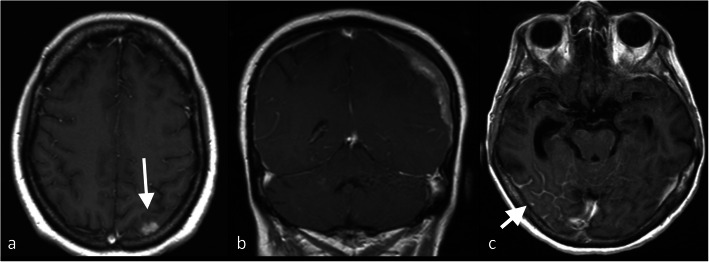
Cerebral manifestations of breast cancer. Post-contrast T1-W MRI in a 58-year-old woman with breast cancer demonstrates intracerebral metastases (**a**), dural metastasis (**b**) and leptomeningeal disease (**c**)

For the detection of brain metastases, contrast-enhanced MRI has higher sensitivity than CT [72], due to its higher soft tissue resolution and its better detection of parenchymal and leptomeningeal involvement [21]. Lesions are usually supratentorial, and they can be solitary or multiple, arising at the grey-white matter junction and at watershed zones of major arterial territories [73] (Fig. 5). Moreover, MRI showed higher sensitivity and specificity than PET-CT for brain metastases [74] as the high background activity present in the cortex and basal ganglia (due to intrinsic high glucose consumption of these structures) can substantially degrade signal-to-noise ratio of FDG PET [72], therefore adversely affect the ability of PET to detect especially small metastatic lesions.

Nevertheless, according to the updated ASCO’s guidelines, clinicians should not perform routine MRI to screen all the breast cancer patients for brain metastases but only in patients with HER2-positive advanced breast cancer because their high incidence of brain metastases [75].

Lungs are frequently involved by breast cancer metastases: with advanced MR techniques, lung metastases may be visualised as foci of high signal on DWI [10].

### Advanced imaging

Among the various imaging modalities currently available for distant metastases detection, hybrid techniques which fuse morphological and functional data are the most sensitive and specific, and PET-CT and PET-MRI will probably continue to evolve and become increasingly important in this field. Different imaging modalities are often used in combination to optimally detect bone metastases. Although conventional imaging has frequently been used for cancer staging, these methods have been found to be less sensitive and less specific in providing accurate data in a clinically relevant time frame. Owing to positive recent developments in advanced imaging, the current trend is toward whole-body imaging in a single session. The choice of modality is usually based on the clinical situation and the type of primary tumour. Further research is warranted to further address the impact of these costly and labour-intensive imaging methods on treatment strategies and on the course of illness. Below, we discuss recent evidences on advanced imaging methods including PET-CT, PET-MRI and WBMRI. These modalities generally show improvements in diagnostic accuracy for detection of metastases over conventional imaging methods, with the ability to quantify biological processes related to the bone microenvironment as well as tumour cellularity. Comparative studies between conventional and advanced imaging techniques have also been carried out in subjects selected for further investigation after bone scan, and the contribution of hybrid imaging and WBMRI to evaluate bone/visceral lesions or suspicious symptoms have been reported [67].

### Hybrid imaging

The use of hybrid imaging (PET-CT and PET-MRI) has increased thanks to the increased access to commercial radiopharmaceutical production facilities and to the widespread availability of scanners [76]. The net clearance of 18F-sodium fluoride (^18^F-NaF) in breast cancer bone metastases is 3–10 times greater than that in healthy bone, conferring the capacity to detect both osteolytic and osteosclerotic metastases [77]. The mechanism of uptake of 18F-NaF tracer into bones is like that of ^99m^Tc diphosphonates, being related to local blood flow and osteoblastic activity, with rapid initial uptake into bone mineral as fluorapatite. ^18^F-NaF PET-CT has better diagnostic performance than ^18^F-NaF PET alone without the CT component [78].

**Fig. 6:**
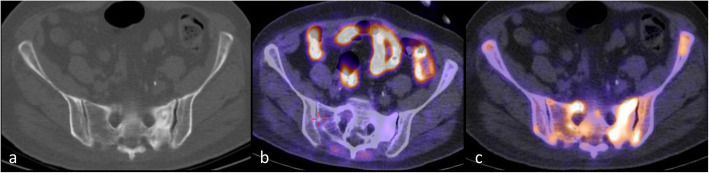
Comparison of NaF and FDG PET-CT In the same 65-year-old woman with metastatic breast cancer a CT (**a**) shows increased sclerosis in the left sacrum, increased tracer uptake of NaF PET in left sacrum and also in both ilium (**b**) but disease activity less well seen on FDG PET (**c**)

In oncological staging, 18F-fludeoxyglucose (^18^F-FDG) is the most widely used radiotracer [76]: its uptake in skeletal metastases is pretended to be largely within tumour cells acting as a breast cancer-specific tracer rather than reflecting alterations in the bone microenvironment [79] (Fig. 6).

The Royal College of Radiologists (UK) recommends the consideration of PET-CT for staging breast cancer patients in those with multifocal disease, suspected recurrence when breast MRI is not possible and in symptomatic patients with an equivocal MRI. In addition, breast cancer patients under the age of 40 may benefit from PET-CT in early disease as this may result in upstaging of systemic sequelae [19].

The underlying histologic subtype of breast cancer may also influence the choice of PET imaging. Untreated invasive lobular carcinoma may have poorer ^18^F-FDG uptake of the osteoblastic metastases compared with invasive ductal or mixed subtypes [80]. Similarly, previous treatment history is significant, as ^18^F-FDG–negative skeletal metastases may show increased sclerotic following successful systemic therapy, which renders tumour cells nonviable even though ongoing reparative osteoblastic activity, as detected by BS or ^18^F-NaF PET may endure [81].

#### PET-CT

There is increasing evidence that PET-CT detects distant metastases with higher sensitivity than conventional imaging [82], although in cases of lobular cancers and low-grade tumours, PET-CT may be less sensitive [83].

In 2012, Brennan and Houssami [84] published a systematic review of studies evaluating the accuracy of imaging for detecting asymptomatic metastases in 14,824 patients with breast cancer. They showed a 7.0% (1.2–48.8%) prevalence of distance metastases, and the following diagnostic sensitivity/specificity: combined conventional imaging 78.0%/91.4%; BS 98.0%/93.5%; CT chest/abdomen 100%/93.1%; FDG-PET 100.0%/96.5%; PET-CT 100%/98.1%, respectively. Accordingly, the performance of both conventional and advanced imaging was very high, but these findings could have been biased by patient selection in each category: in comparative studies, PET-CT had significantly higher sensitivity (98.7%, 78–100%) than conventional imaging (70%, 37.5–85.9%).

Minamimoto et al. [85] prospectively evaluated combined ^18^F-NaF and ^18^F-FDG PET-CT in 15 women with breast cancer and 15 men with prostate cancer and compared the results with those of BS and WBMRI (including DWI). For bone metastases, PET-CT demonstrated better sensitivity and accuracy than WBMRI (96.2% vs. 81.4%, 89.8% vs. 74.7%,) and BS (96.2% vs. 64.6%, 89.8% vs. 65.9%,), while, for visceral metastases, ^18^F-NaF/^18^F-FDG PET-CT and WBMRI had no statistically significant difference in sensitivity, PPV or accuracy.

The higher lung tissue contrast of the CT and its lower susceptibility to motion artefacts facilitate the detection of lung lesions [68] and, some authors support PET-CT as currently the best advanced imaging method for detecting lung metastases, with a sensitivity of 89% for PET-CT compared to 82% for WBMRI and 74% for PET-MRI [86, 87].

Additionally, PET-CT is useful for the detection of metastatic mediastinal and supraclavicular lymph nodes and a recent meta-analysis [88] reported a sensitivity, specificity, positive predictive value, negative predictive value and accuracy for detecting nodal spread of 84%, 92%, 74%, 82% and 81%, respectively. According to Schmidt et al. [89], PET-CT appears to have an increased specificity for neoplastic axillary and mediastinal lymph nodes compared to WBMRI, namely 90% versus 86%.

#### PET-MRI

The combination of PET with MRI may benefit from the high soft tissue contrast of MRI with the additional use of multi-parametric MRI. Moreover, PET-MRI systems overcome pitfalls of PET-CT, such as uptake in bowel loops and ureters, which may lead to false positive findings [90].

However, while claustrophobia is not an issue with PET-CT, many patients do experience it with PET-MRI due to a combination of the relatively narrower and longer bore and the use of whole-body anterior surface coils and a head coil required for the MRI portion of the exam [91]. Moreover, PET-CT protocols are usually completed within 30 min, which are faster than PET-MRI that is designed to take advantage of multi-sequences MRI, resulting in studies that may exceed an hour in length with discomfort of many cancer patients who are unable to remain supine and still for long time due to pain [91]. Although some authors proposed faster and abbreviated PET-MRI protocols [92–95] that allow PET-MRI to rival PET-CT for speed, such protocols often do not take full advantage of PET-MRI and may fail to justify the additional expense of PET-MRI.

Tumours that are assessed by both PET-CT and MRI could be transferred to PET-MRI [76] as it allows combined local and whole-body staging in breast cancer, providing an improved lesion detection in the brain, breast, liver, kidneys and bones. Indeed, MRI can provide an anatomic correlate for an FDG-avid lesions and it can detect bone metastases with low FDG uptake (which are not detectable on PET-CT) [82, 96]. According to Melsaether et al. [87], PET-MRI outperformed PET-CT in the detection of metastatic breast cancer and it showed, at the same time, a dose reduction averaging of 50%. Even though PET-CT detects more pulmonary lesions than PET-MRI, the clinical significance of lung lesions missed on PET-MRI is unclear [97]. On the other hand, Riola-Parada et al. [98] in their systematic review of 57 articles evaluating the PET-MRI versus PET-CT in the metastases detection showed a similar diagnostic performance between the two modalities except for small lung metastases, for which PET-CT was superior.

Although PET-CT has the advantage in terms of overall acquisition time due to the rapid nature of CT scanning, PET-MRI may acquire higher quality PET images due to longer time available for acquisition and the ability to use MRI respiratory gating information to improve PET data [91]. Usually, MRI exams are obtained in the anatomic target/region of interest and hence extra time spent at this bed position permits a longer PET acquisition providing increased detector counts and improved image quality. Moreover, because MRI sequences are typically obtained during breath-holds or using respiratory gating, PET data can be reconstructed with selected data at motion free time points, allowing for decrease in motion artefact [91].

In conclusion, PET-CT is currently an established technology already in widespread and accepted use worldwide, while PET-MRI has higher cost and major complexity of operating and interpreting the findings. However, PET-MRI can decrease radiation dose and improve motion correction [91].

#### Whole-body MRI

The introduction of DWI and apparent diffusion coefficient (ADC) improved the accuracy of MRI for metastases detection [99–101]. Using whole-body DWI with STIR fat suppression in free breathing optimises the image signal-to-noise ratio and image quality [102, 103]. In addition to axially acquired DWI, T1WI, STIR and/or T2-weighted fat-suppressed images are usually also obtained [104]. Regarding the choice of *b* value, at least two *b* values are suggested: the higher *b* value chosen usually ranges between 600 and 1000 s/mm^2^, while the lower b value ranges between 0 and 100 s/mm^2^ [102]. The protocol currently used for WBMRI is feasible, reproducible and can be performed in a relatively short time [10].

In metastatic breast cancer, DWI appears equally sensitive but less specific than 19F-FDG PET-CT in the bone metastases evaluation, indicating that it should not be read in isolation but in correlation with morphologic imaging [105]. In 2010, a prospective study [106] enrolling 36 patients with prostate or breast cancer showed a better performance of WBMRI in detecting malignant skeletal lesions (sensitivity 97% vs. 91%) compared to BS (sensitivity 97% and 48%, respectively). The recent SKELETA trial [124] compared the diagnostic accuracy of BS, SPECT, SPECT-CT, ^19^F-NaF PET-CT and WBMRI for the detection of bone metastases in 26 breast and 27 prostate cancer patients, reporting the following sensitivity values, 62%, 74%, 85%, 93% and 91%, respectively, resulting in an analogous diagnostic accuracy to 19F-NaF PET-CT and outperformed SPECT-CT and BS.

Yang et al. [128] performed a meta-analysis in several tumour types, including breast cancer, and showed that WBMRI and PET-CT resulted superior to CT and BS in terms of both sensitivity and specificity to detect bone metastases. Particularly, the sensitivity rates for PET-CT, CT, MRI and BS were 89.7%, 72.9%, 90.6% and 86.0%, respectively, and the specificity rates were 96.8%, 94.8%, 95.4% and 81.4%, respectively. Results of singular studies are showed in Table 3.

**Table 3:** Studies comparing diagnostic performance of WBMRI and PET-CT

Ref	First author	Year	Population	Use of DWI	Sensitivity of WBMRI (vs. PET-CT)	Specificity of WBMRI (vs. PET-CT)	Accuracy of WBMRI (vs. PET-CT)	Comments
[9]	Jacobs	2018	22 patients with stage IV	Yes	96% (vs. 80%)	N/R	N/R	ADC values were significantly increased in bone lesions while they were decreased in soft tissue metastases.
[107]	Schmidt	2007	30 patients with different stages	Yes	94% (vs. 78%)	76% (vs. 80%)	91% (vs. 78%)	WBMRI showed superior accuracy in bone marrow screening compared to PET-CT.
[86]	Antoch	2003	98 patients with different stages	No	90% (vs. 93%)	95% (vs. 95%)	93% (vs. 94%)	PET-CT showed better performance. However, the WBMRI was performed without DWI.
[89]	Schmidt	2008	33 patients with breast cancer and suspicious of recurrence	Yes	93% (vs. 91%)	86% (vs. 90%)	91% (vs. 91%)	It was also assessed that staging with WBMRI is feasible at 1.5 and 3 T, noting that scan time is reduced at 3 T with identical resolution.

The reasons for false positive results on WBDWI include bone marrow oedema caused by benign conditions, which can be overcome by correlating the high *b* value DWI with related ADC maps and conventional MRI sequences based on T1W.

A number of studies translationally analysed the differences between normal bone marrow and metastatic lesions, correlating ADC with cellularity and other histological characteristics in bone metastases [58, 108, 109]: this is essential for qualification of MRI as a prognostic biomarker in metastatic breast cancer [17, 20, 21, 52].

**Fig. 7:**
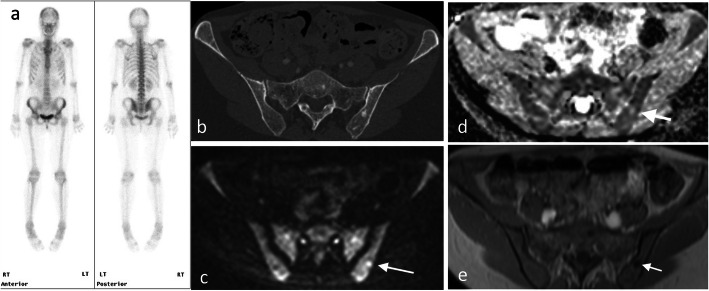
Evaluation of activity of bone disease on bone scan versus WB-MRI with diffusion. Bone scan (**a**), CT (**b**) and WBMRI with diffusion (**c** and **d**) and T1-W (**e**) in a 55-year-old woman with breast cancer post-treatment. The bone scan shows no significant abnormality (**a**), the CT shows foci of bone sclerosis in the pelvis with a visible lesion in the left posterior ilium (**b**). The lesions demonstrate low signal on the T1-W image (**e**) and high signal on DWI and intermediate ADC in keeping with disease

**Fig. 8:**
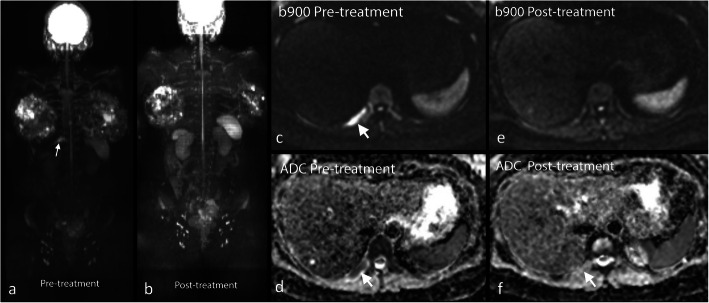
WBDWI in bone disease following chemotherapy. WB-MRI in a 59-year-old woman with breast cancer. 900 MIP before (**a**) and after (**b**) chemotherapy as well as b-900 and ADC maps pre (**c** and **d**) and post (**e** and **f**) chemotherapy demonstrate a right 12th rib lesion on with high DWI signal and low ADC on the pretreatment study (white arrow **d**) and complete response of the lesion after treatment (**e** and **f**)

In addition, WBMRI can play a role in the assessment of visceral metastases [62, 110], where WBMRI has a better sensitivity than PET-CT to evaluate small hepatic and brain metastases (Fig. 7) [69, 89]. There is also an evolving role for the use of WBDWI to assess the treatment response of metastatic bone disease to systemic and targeted therapies (Fig. 8).

### Future perspectives

Nowadays, the processes of breast cancer metastasis have been well characterised at the molecular level and numerous biomarkers of tumour aggressiveness have been discovered and are ready to be tested in clinics. As we have discussed, molecular imaging offers the opportunity to depict specific cell markers relevant to tumour aggressiveness. For instance, Harmon et al. [111] recently compared in a prospective way the ^18^F-NaF PET-CT with ^18^F-N-[N-[(S)-1,3-dicarboxypropyl]carbamoyl]-4-F-fluorobenzyl-L-cysteine (DCFBC) PET-CT in patients with prostate cancer. Interestingly, this study appears to show that such tracer uptake depends on the disease course and treatment status, which may indicate a functional difference in bone metastases. The study showed that ^18^F-DCFBC PET-CT detected significantly less bone lesions, especially in patients who were found to be castrate sensitive. On the other hand, in more advanced disease, there was good concordance between the two radiotracers. ^18^F-DCFBC, though, demonstrates a high blood-pool uptake and such second-generation tracer may elucidate more functional information on the pathophysiology of bone metastases.

Although target-specific molecular imaging probes for tumour invasiveness have been developed for PET (i.e. proteases associated with tumour invasion, such as specific matrix metalloproteinases or cathepsins, can be targeted ‘*in vivo’* with PET), they have not yet been widely used with MRI [112]. Novel MRI contrast agents based on iron oxide and dendrimer nanomaterials allow for better characterisation of tumour metastases [112]. Particularly, ultrasmall superparamagnetic particles of iron oxide (USPIOs) imaged with MRI does not require ionising radiation, yet can detect small metastases [113]. The rationale for the use of USPIO is that, after intravenous injection, the nanoparticles are phagocytosed by macrophages in circulation which then enter the interstitial space and are taken up by lymphatics [114–117]. Since iron oxide is superparamagnetic, it becomes strongly magnetic in the strong magnetic field of the MRI leading to spin dephasing and susceptibility effects which result in signal loss. Therefore, the signal intensity is markedly reduced in healthy tissues due to the magnetic susceptibility and T2WI shortening effects of the USPIO particles [114, 118]. Conversely, in areas of metastases, there is much less uptake of USPIO particles and, therefore, those portions of the LNs remain unchanged in signal on T2WI 24–48 h after intravenous injection of a USPIO [114–117]. Current limits of USPIOs’ use include the difficulty of image acquisition/interpretation and the lack of approved USPIOs themselves which are clinically available hinders adoption and larger studies.

Organ-specific MRI contrast agents are also used to identify metastatic disease in the liver. Superparamagnetic iron oxide particles (SPIO) have been approved for clinical practice use as a ‘negative contrast agent’ for the normal liver parenchyma in order to visualise benign and malignant hepatic cancers [119]. Dendrimer-based macromolecular MRI contrast agents as diaminobutane (DAB) dendrimers, that differ from polyamidoamine (PAMAM) dendrimers because they have a pure aliphatic polyamine interior, homogeneously enhance the liver parenchyma and are excreted more rapidly through both the liver and kidney than the analogous PAMAM dendrimer of similar molecular size [120]. Kobayashi et al. were able to detect small metastatic lesions on the liver from colon cancer cells using DAB dendrimers [120]. Additionally, gadolinium ethoxybenzyl diethylenetriaminepentaacetic acid (Gd-EOB-DTPA) has been developed as a liver-specific contrast agent by a small modification of conventional gadolinium-based chelates. As Gd-EOB-DTPA is taken up by hepatocytes, the normal liver parenchyma can be visualised by increased signal intensity at *T*1WI, generating a positive contrast enhancement, where normal liver parenchyma is bright on T1WI and the liver metastases are shown as focal areas of relative hypointensity [121].

Finally, hybrid imaging techniques of combining WBMRI with ^18^F-choline PET have recently showed to have a significantly higher sensitivity (93.5%) when compared to BS (63.6%) and WBMRI on its own (72.7%) [122]. The results are promising, but with limited access to PET-MRI and its high cost, a defined role of PET and newer advanced hybrid imaging techniques are needed [113]. Stratifying patients according to disease course and treatment status may prove beneficial in the future. Nevertheless, a hybrid role of functional imaging provided by biomarkers with the anatomical detail provided by imaging techniques will offer a valuable insight into disease status.

## Limitations

Substantial heterogeneity was evident in the published literature that were reviewed. Firstly, the quality of the reference standard was variable, relying on combinations of biopsy and/or follow-up. Secondly, there was also variability in the prevalence of bone and visceral metastases across studies. Moreover, we observed that most studies did not quantify how detection of metastases, especially when asymptomatic, influenced the management. As information on treatment and survival were not integrated into such studies, it is not possible to infer the likely prognostic implications of the imaging performance in this patient cohort.

## Conclusions

Accurate M-staging is crucial in the selection of the most appropriate treatment for patients with advanced breast cancer. Although CT, BS and conventional MRI are still the most widely used imaging modalities in this disease setting, advanced imaging techniques are increasingly employed for the earlier and more accurate detection of metastatic disease, for both bones and visceral disease.

## Data Availability

Data originating the tables are available on request sent to the corresponding author.

## Abbreviations

BM: Bone metastases
BS: Bone scintigraphy
CT: Computed tomography
DWI: Diffusion-weighted imaging
ER/PR: Oestrogen/progesterone receptor
MRI: Magnetic resonance imaging
NCCN: North-American National Comprehensive Cancer Network
PET: Positron-emission tomography
PPV: Positive predictive value
SPECT: Single-photon emission computerised tomography
WBMRI: Whole-body magnetic resonance imaging
WI: Weighted imaging
